# Novel Technologies for Preserving Ricotta Cheese: Effects of Ultraviolet and Near-Ultraviolet–Visible Light

**DOI:** 10.3390/foods9050580

**Published:** 2020-05-05

**Authors:** Emilio Francesco Ricciardi, Selene Pedros-Garrido, Kostas Papoutsis, James G. Lyng, Amalia Conte, Matteo A. Del Nobile

**Affiliations:** 1 Department of Agricultural Sciences, Food and Environment, University of Foggia, Via Napoli, 25-71122 Foggia, Italy; ricciardifrancesco84@gmail.com (E.F.R.); matteo.delnobile@unifg.it (M.A.D.N.); 2 School of Agriculture and Food Science, University College Dublin, Belfield, 4 Dublin, Ireland; selene.pedros-garrido@ucd.ie (S.P.-G.); kostas.papoutsis@ucd.ie (K.P.); james.lyng@ucd.ie (J.G.L.)

**Keywords:** UV-C, NUV–vis light, non-thermal technology, irradiation treatments, microbial inactivation, ricotta cheese

## Abstract

Ricotta cheese is a potential growth medium for a wide range of microorganisms. The aim of the current study was to investigate the efficacy of ultraviolet (UV-C) and near-ultraviolet–visible light (NUV–vis) in microbial decontamination of ricotta artificially inoculated with *Pseudomonas fluorescens*. Cheese samples were stored at 4 °C, and microbiological and sensory analyses were performed for 9 days. From the microbiological point of view, control samples became unacceptable after less than 5 days, whereas ricotta treated by both UV-C and NUV–vis light remained acceptable for more than 6 days. Similar effects of UV-C and NUV–vis light were also recorded in terms of sensory quality. The shelf life of the samples subjected to the treatments was thus extended by 50%, suggesting the potential application of UV-C and NUV–vis light for cheese decontamination.

## 1. Introduction

Ricotta cheese is traditionally prepared by heating whey and acidifying the resultant hot liquid with lactic acid to coagulate whey proteins [1,2]. Ricotta cheese is an excellent growth medium for a wide range of microorganisms, mainly represented by *Pseudomonas* spp., yeasts, molds, and Enterobacteriaceae [3]. *Pseudomonas* spp. represent the limiting microorganism group for ricotta shelf life, and with levels over 7 log colony-forming units (CFU)/g, the product is considered spoiled [4]. Spoilage of fresh ricotta caused by *Pseudomonas* spp. could provoke an important color change due to the secretion of yellow-green pyoverdine and blue pigments [5,6]. The cooling phase of heat-treated ricotta cheese is a likely stage where re-contamination could occur. In particular, the temperature range from 40 to 10 °C is favorable for *Bacillus cereus* growth [7,8].

In order to control the initial microbial load, the rigorous application of good hygiene and good manufacturing practices is essential [9]. Depending on the food industry operator, fresh ricotta cheese can be packaged in air or undergo modified atmosphere packaging (MAP) (30% CO_2_ and 70% N_2_) [10], leading to a shelf life between 7 and 21 days. On the other hand, the industrial process that involves a final thermal treatment step guarantees shelf life duration between 20 and 40 days [3].

In order to obtain an appropriate shelf life duration, overcome the limitations of thermal sterilization, and ensure food safety and quality, with a minimal impact on food nutritional properties and on the environment, alternative non-thermal treatment technologies are continuously being investigated [11,12].

The use of ultraviolet light (UV) as a decontamination technology is very promising, having the advantage of being a cheap method [13,14,15]. The UV region is traditionally divided in three wavelength spectra, UV-A (315–400 nm), UV-B (280–315 nm), and UV-C (200–280 nm). Microbial inactivation produced by UV-C is mainly due to DNA damage (e.g., pyrimidine dimers formation), which inhibits transcription and replication and can lead to cell death. Moreover, this treatment leads to minimal loss of nutritional and sensory quality of foods, and no toxic effects or residues are associated with UV treatment [16].

Light-emitting diodes (LEDs) are an alternative source of ultraviolet light, also suitable for applications in the food industry [17]. The cost of LEDs, which have a compact and robust design, has recently decreased, substantially due to technological advances. The output power is becoming increasingly efficient. Furthermore, the average duration of LEDs, compared to UV lamps currently used in the industry, is longer, and LEDs’ heat emissions are lower than those of UV lamps. Therefore, LEDs are more suitable for food processing applications [18]. The blue region of the visible light spectrum (400–495 nm), and specifically the near-ultraviolet–visible light (NUV–vis) (390–400 nm), has a greater effect on microbial inactivation compared to other regions [19]. As the LED technology is environmentally friendly, energy-efficient, safe, and long-lasting, LED-based photodynamic inactivation is increasingly explored in food processing [20,21]. The photo-inactivation of microbes is attributed to the production of reactive oxygen species (ROS) by intracellular photosensitizer (PS) excitation; in turn, ROS can interact with nucleic acid bases of the DNA, unsaturated fatty acids, and amino acid residues, eventually causing bacterial death [17].

Considering the potential as bactericides of UV-C and NUV–vis light, the aim of this study was to assess their effect on ricotta cheese inoculated with *Pseudomonas fluorescens*. Therefore, during a period corresponding to ricotta shelf life, microbiological and sensory parameters were evaluated.

## 2. Materials and Methods

### 2.1. Sample Preparation

Ricotta cheese (2.5 kg) was purchased at a local supermarket (Tesco, Dublin, Ireland) and transported to the laboratory under refrigerated conditions. The ricotta was manipulated under aseptic conditions to prepare the experimental samples. Cheese was placed into small Petri dishes (diameter 5.5 cm, thickness 1.3 cm, surface 57 cm^2^, volume 30.87 cm^3^, surface/volume (S/V) ratio 1.84 cm^−1^, net weight 24.24 g) in order to use a container with a high surface/volume ratio and increase the efficacy of the two non-thermal treatments to be applied.

Two tests were made: a preliminary test to study the influence of bacterial concentration on the decontaminating effect of UV-C and NUV–vis and a subsequent shelf life test to assess the effect of the two technologies on the quality of bacteria-inoculated ricotta during storage.

The preliminary test was performed with a total of 12 ricotta samples, differently inoculated and then treated by UV-C and NUV–vis, subjected to MAP (30%CO_2_/70%N_2_) using a tray sealer (Ilpra, Cheshire, UK), and stored at 4 °C. Microbial enumeration after treatments was carried out to evaluate the inactivation level.

For the shelf life study, a total of 36 ricotta samples were inoculated at a specific microbial inoculum concentration, treated by UV-C and NUV–vis, subjected to MAP (30%CO_2_/70%N_2_), stored at 4 °C, and analyzed during the subsequent 9 days for microbiological and sensory quality; individual cheese samples were available at each time point. In both preliminary and shelf life tests, control samples of ricotta (inoculated but not treated) were also considered.

### 2.2. Sample Inoculation

To inoculate the ricotta samples, different concentrations of *P. fluorescens* were used. To prepare the inoculum, a *P. fluorescens* (DSM 50090, type strain, DSMZ-German Collection of Microorganisms and Cell Cultures GmbH) culture was prepared in 10 mL of sterilized Tryptic Soy Broth (TSB, Oxoid, Basingstoke, UK) and then incubated at 25 °C for 24 h. Then, the culture was diluted to adjust the microbial concentration, which was measured with a spectrophotometer (UV-mini 1240, Spectrophotometer, Shimadzu, Duisburg, Germany). In the preliminary test, samples of ricotta were inoculated with 10^5^, 10^4^, and 10^3^ CFU/mL. For the shelf life study, the concentration selected for the inoculums was 10^3^ CFU/mL. For the inoculation, 50 µL of microbial suspension was spread on the product surface, and the suspension was completely absorbed by the cheese. The time between inoculation and treatment was about 1 h for all samples.

### 2.3. UV-C and NUV–Vis Treatment

The samples were treated once, on the same day; this corresponded to time zero of the storage period. In both preliminary and shelf life tests, only one light dose per technology was tested.

The UV unit was custom-made, with internal dimensions of 790 × 390 × 345 mm (L × W × H) and consisted of four 95-W bulbs of 50 cm length (Baro Applied Technology Limited, Manchester, UK). An initial characterization of the UV chamber was performed at a total of 27 carefully selected locations within the chamber, using a radiometer ILT 1700 (International Light Technologies, Boston, MA, USA) connected to a solar blind vacuum photodiode detector (configuration SED240/NS254/W) for measuring the energy delivered at 254 nm. The aim of these measurements was to obtain the actual energy received by the sample, which may vary depending on the sample position within the chamber, its distance from the lamps, and the treatment time. To treat the samples, a specific position to reach a final dose of 6.54 J/cm^2^ (3.5 cm from the lamp for 30 s) was selected.

The NUV–vis light was produced by a LED array (OD-2049) (Opto Diode Corp, sourced from AP Technologies, Bath, UK) with a center wavelength of 395 ± 5 nm, a bandwidth of 12 nm full-width at half maximum (FWHM), and a half intensity beam angle of 30° [19]. The irradiance of light emitted from the LED unit (J/cm^2^) was measured using a UV–VIS radiometer (model no. RM12, Dr. Gröbel UV Electronik, GmbH, Ettlington, Germany) fitted with an RM UV-A sensor (part no. 811030, Dr. Gröbel UV Electronik) at different locations within the unit. Based on the equipment characterization results, all treatments of the samples were performed in a central position, at 6 cm from the LED light source (perpendicular light incidence) for 400 s, applying a total dose of 6.36 J/cm^2^.

### 2.4. Shelf Life Study

During shelf life, bacterial enumeration was carried out. To this aim, from each package, 10 g of ricotta was taken, diluted with 100 mL of a 0.9% NaCl solution in a stomacher bag (Blander Blags, Cona, Ferrara, Italy), and homogenized (400 circulators—Seward, Hamilton, Islandia, New York, USA) for 60 s. Subsequently, appropriate decimal dilutions of the homogenates were made for microbial counts, using the same diluent. *Pseudomonas* spp. enumeration was performed on *Pseudomonas* CFC (Cetrimide, Fucidin, Cephalotin) selective agar (Oxoid), after incubating the plates for 48 h at 25 °C. Enterobacteriaceae counts were carried out using VRBGA (violet red bile glucose agar) medium (Aucmedia Lab 088), after incubating the plates for 24 h at 37 °C [22], while Sabouraud Agar (SAB) (Nogen Culture Media NCM 2012A) was used for the detection of yeasts [23]. Mesophilic bacteria were enumerated using PCA (plate count agar) medium (Scharlau), after incubating the plates were for 48 h at 30 °C. Finally, *B. cereus* counts were performed using *B. cereus* Agar Base (PEMBA, Oxoid) culture medium, adding egg yolk emulsion (SR0047, Oxoid) and polymyxin B supplement (SR0099E, Oxoid) [3]. All analyses were performed at least in duplicate for each sample.

In order to calculate the microbial acceptability limit, indicated as MAL, a modified version of the Gompertz equation was used to fit *Pseudomonas* spp. and yeast counts as limiting microbial groups for ricotta shelf life (Equation (1)):

(1) $$\log\left(N\left(t\right)\right)=\log\left(N_{\max}\right)-A\cdot \exp\left\{-\exp\left\{\left[\left(\mu_{\max}\cdot 2.71\right)\cdot \frac{\lambda -\mathrm{MAL}}{A}\right]+1\right\}\right\}+A\cdot \exp\left\{-\exp\left\{\left[\left(\mu_{\max}\cdot 2.71\right)\cdot \frac{\lambda -t}{A}\right]+1\right\}\right\}$$

where N(t) is the viable cell concentration at time t, A is related to the difference between the decimal logarithm of maximum bacteria growth attained at the stationary phase and the decimal logarithm of the initial value of cell concentration, μ_max_ is the maximal specific growth rate, λ is the lag time, N_max_ is the microbial threshold value, MAL is the microbiological acceptability limit (i.e., the time at which N(t) is equal to N_max_), and t is the storage time [3,22]. The value of N_max_ was set to 10^7^ CFU/g for both *Pseudomonas* spp. and yeasts because literature data confirm that this level of contamination is compatible with a possible alteration of the product [9,24]. The goodness of fitting was evaluated by the χ^2^ value.

A sensory screening of ricotta samples was also performed by a group consisting of seven people, members of the laboratory of the School of Agriculture and Food Science of the University College of Dublin. Panelists were asked to judge odor, color, consistency, and overall quality, using a 7-point scale. The score of 4 was set as the minimum threshold for product acceptance [25]. Analyses were performed at least in duplicate for each sample. The sensory acceptability limit, indicated as SAL, was calculated in order to establish the remaining number of days the cheese was sensory-acceptable [3,22]. To this aim, the same modified version of the Gompertz equation was used to fit the sensory experimental data, by setting the score 4 as the threshold for product acceptability (Equation (2)):

(2) $$\mathrm{OSQ}\left(t\right)={\mathrm{OSQ}}_{\min}-A^{Q}\cdot \exp\left\{-\exp\left\{\left[\left(\mu_{\max}^{Q}\cdot 2.71\right)\cdot \frac{\lambda^{Q}-\mathrm{SAL}}{A^{Q}}\right]+1\right\}\right\}+A^{Q}\cdot \exp\left\{-\exp\left\{\left[\left(\mu_{\max}^{Q}\cdot 2.71\right)\cdot \frac{\lambda^{Q}-t}{A^{Q}}\right]+1\right\}\right\}$$

where OSQ(t) is the ricotta overall sensory quality at time t, A^Q^ is related to the difference between the ricotta overall quality attained at the stationary phase and the initial value of ricotta quality, μ_max_ is the maximal rate at which OSQ(t) decreases, λ^Q^ is the lag time, OSQmin is the threshold for sensory acceptability, SAL is the sensory acceptability limit (i.e., the time at which OSQ(t) is equal to OSQmin), and t is the storage time. The goodness of fitting was evaluated by the χ^2^ value.

For shelf life estimation, it was considered that wherever the global quality of a food product depends on several quality sub-indices, its shelf life is, by definition, the time at which one of the product quality sub-indices reaches the threshold. Therefore, in the current study, the shelf life of each tested sample was calculated as the lowest value among the three fitting parameters, i.e., the two MAL values and the SAL value.

### 2.5. Experimental Plan and Statistical Analysis

Tests were carried out on duplicate batches. Experimental data are the average of two replicates. Fitting parameters were compared by one-way ANOVA. A Duncan’s multiple range test, with the option of homogeneous groups (*p* < 0.05), was used to determine significance among differences. All these analyses were performed with Statistica 7.1 for Windows 152 (StatSoft Inc., Tulsa, OK, USA).

## 3. Results and Discussions

### 3.1. Effects of UV-C and NUV–Vis Light Depending on the Contamination Level

**Figure 1:**
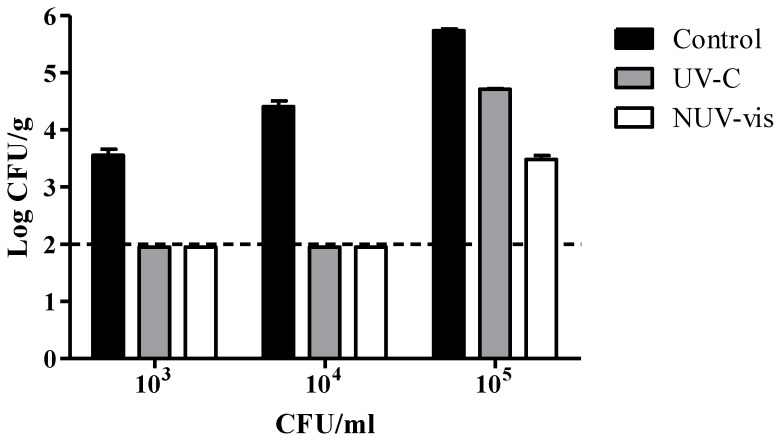
Mean log colony-forming units (CFU)/g of *Pseudomonas* spp. recorded in the preliminary test in ricotta cheese inoculated with three different concentrations (10^3^, 10^4^, and 10^5^ CFU/mL) of *Pseudomonas fluorescens* (DSM 50090) immediately after treatments with ultraviolet light (UV-C) and near-UV–visible light (NUV–vis) at one light dose per technology, with their respective controls (inoculated and not treated). A total of 12 ricotta samples were treated once, on the same day, with UV-C and NUV–vis. The dashed horizontal line represents the detection limit (≥100 CFU/g). Each bar represents mean ± SD.

In the preliminary test, prior to the shelf life study, ricotta samples inoculated with different concentrations of *P. fluorescens* (i.e., 10^3^, 10^4^, and 10^5^ CFU/mL) were exposed to UV-C and NUV–vis treatments. Thus, the influence of the bacterial concentration on the decontaminating effect of both technologies was assessed. The inactivation levels of *Pseudomonas* spp. after these treatments are shown in Figure 1. *Pseudomonas* spp. levels in cheese after inoculation with 10^3^, 10^4^, and 10^5^ CFU/mL were 3.6 ± 0.1, 4.4 ± 0.1, and 5.7 ± 0.03 CFU/g, respectively. Exposure to UV-C and NUV–vis light caused a decrease of the microbial population below the detection limits (<100 CFU/g) when 10^3^ and 10^4^ CFU/mL were used as inoculation levels. When 10^5^ CFU/mL were inoculated, the inactivation levels were −1.03 ± 0.02 and −2.26 ± 0.04 CFU/g after UV-C and NUV–vis treatments, respectively. These results coincide with those of other authors who also found that the effectiveness of ultraviolet light depends on several factors, such as the characteristics of species and bacterial strains, growth rate, initial bacterial population density, method of inoculation, composition, and food type [26]. Specifically, the method of inoculation and the concentration of cells added can affect the inactivation efficiency when light treatments are used, due to the formation of agglomerates, clusters, or layers of bacterial cells that cause a shadowing effect, impeding light penetration and bacterial inactivation [27,28]. Furthermore, it is also important to consider that ricotta is a food matrix with surface irregularities, which may act as physical protection against light, contributing to bacterial survival. At a microscopic level, the surface of ricotta is not smooth but characterized by roughness, which is expected to shade microbial cells, further compromising the germicidal effect of light [22].

It has also been demonstrated that UV-C light can promote protein degradation, thus increasing nutrient bioavailability for the remaining bacteria [29,30]. All these reasons suggest that the application of doses (J/cm^2^) higher than those required for microbial inactivation does not increase the decontamination efficiency.

Therefore, considering this set of factors, the inoculum concentration for the subsequent shelf life test was that with the lowest initial bacterial population density.

### 3.2. Shelf Life Study of Ricotta Cheese Treated with UV-C and NUV–Vis Light

During shelf life, Enterobacteriaceae, *Pseudomonas* spp., yeasts, mesophilic bacteria, and *B. cereus* were monitored in all treated and untreated samples. *B. cereus* was not detected in any of the samples during shelf life, as also reported by Ricciardi et al. [3], who studied the effects of X-ray irradiation on ricotta cheese shelf life. Enterobacteriaceae counts increased from values below the detection limit to 3 log CFU/g in the control cheese and to 2 log CFU/g in the treated samples. The experimental findings recorded for treated cheese are supported by the literature, because also the study of Allende et al. [31] confirmed that Enterobacteriaceae are more photosensitive than other bacteria.

**Figure 2:**
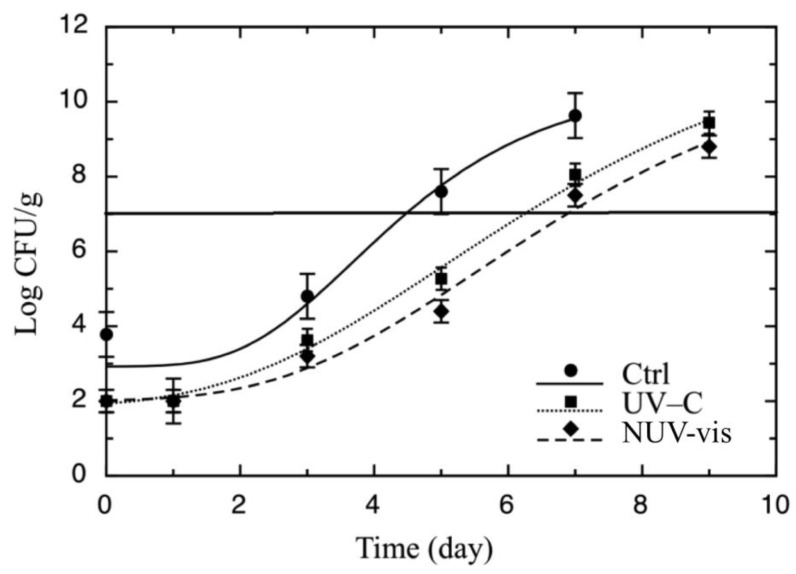
Evolution of *Pseudomonas* spp. during the shelf life test in ricotta cheese inoculated with *P. fluorescens* (DSM 50090) (10^3^ CFU/mL), treated with UV-C and NUV–vis light, at one light dose per technology, with respective control samples (Ctrl, inoculated, and not treated). A total of 36 samples were treated once, on the same day, which corresponded to time zero of the storage period, and then analyzed for 9 days. The continuous line represents the best fitting of Equation (1) to the experimental counts of *Pseudomonas* spp. The horizontal line represents the threshold for microbiological acceptability set to 7 log CFU/g.

Figure 2 shows counts of *Pseudomonas* spp. recorded in ricotta samples treated with UV-C and NUV–Vis light with respect to controls (untreated) during shelf life. Similar to the results found in the preliminary study, the initial level of *Pseudomonas* spp. before treatments was 3.74 ± 0.08 log CFU/g, and after both treatments the microbial levels were below the detection limit. Over the shelf life, these values remained between 1 and 3 log below the control, and after 5 days the levels were 5.24 ± 0.14 and 4.40 ± 0.70 log CFU/g for UV-C and NUV–vis, respectively, while for the control, they were 7.55 ± 0.10 log CFU/g. Therefore, while the untreated samples reached the threshold after 4 days, treated ricotta remained acceptable for more than 6 days. Manzocco at al. [32] also proved the efficacy of UV-C light on *Pseudomonas* spp. in fresh-cut pineapple; they observed great effects of treatment during the first days of storage; however, over the shelf life, microbial counts of all treated samples approached those recorded in the control fruit.

As reported in the Materials and Methods section, microbiological data related to *Pseudomonas* spp. were fitted by a mathematical approach (Equation (1)) to calculate the remaining number of days the cheese was microbiologically acceptable [3,22]. The re-parameterized form of the Gompertz equation is useful for the purpose of the present study. It contains, among the parameters, the MAL value, which represents the number of days necessary to reach the limit that we established (10^7^ CFU/g). Data of the fitting procedure are reported in Table 1. Comparing the results of MAL for *Pseudomonas* spp. of the treated samples reported in the Table, similar values were recorded for UV-C light and NUV–vis light, thus suggesting that both technologies exerted comparable effects against *Pseudomonas* spp., which was also found in our preliminary test. Other studies carried out with different dairy products treated by surface-acting technologies recorded similar effects on *Pseudomonas* spp. For example, Lacivita et al. [22] assessed the effect of UV-C light to control surface contamination and extend the shelf life of Fiordilatte cheese. They inoculated the ‘pasta filata’ cheese with *Pseudomonas* spp. and then exposed it to 0.1, 0.6, 1.2, and 6.0 kJ/m^2^ of UV-C light, packaged it with brine, stored it at 9 °C, and analyzed it for microbial growth and sensory quality. A germicidal effect between 1 and 2 log cycles was observed during storage. In this study, penetration depth was also measured by a luminometer equipped with a UV-C light probe. A very low UV-C light transmittance through the food tissue and an antimicrobial effect limited to a very thin surface layer of the product were found in that study. Similarly, in the current research a surface action of UV radiation was hypothesized.

Hyun and Lee [26] investigated the effect of 460–470 nm light-emitting diode illumination (LED460–470 nm) against pathogenic and spoilage bacteria on the surface of agar media and packaged sliced cheese. Among spoilage bacteria, inoculated *P. fluorescens* showed a high inhibition after treatments. LED460–470 nm treatments were performed at 4 and 25 °C, and microbial reduction levels were higher at 4 °C than at 25 °C, because at lower temperatures cellular injury, cell membrane disruption, and loss of cytoplasmic components were observed.

**Figure 3:**
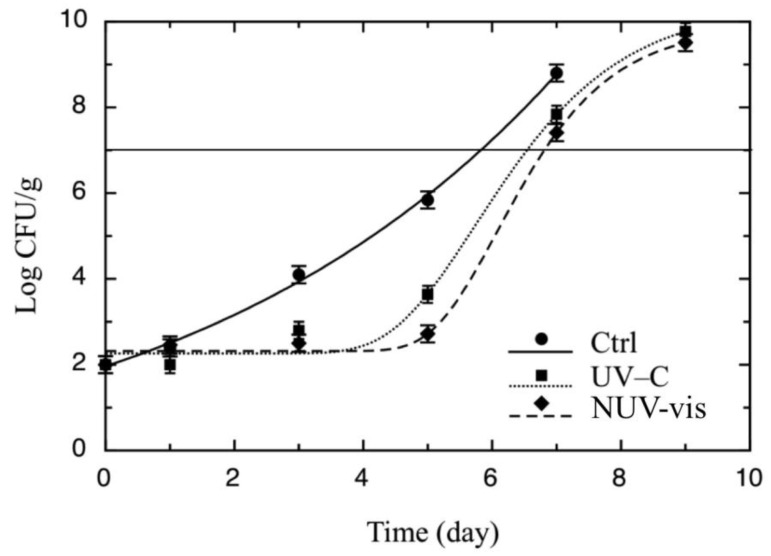
Evolution of yeasts during the shelf life test in ricotta cheese inoculated with *P. fluorescens* (DSM 50090) (10^3^ CFU/mL), treated with UV-C and NUV–vis light, at one light dose per technology, with respective control samples (Ctrl). A total of 36 samples were treated once, on the same day, which corresponded to time zero of the storage period, and then analyzed for 9 days. The continuous line represents the best fitting of Equation (1) to the experimental counts of yeasts. The horizontal line represents the threshold for microbiological acceptability, set to 7 Log CFU/g.

Figure 3 represents yeast counts over the shelf life for treated and untreated cheese samples. Yeast populations grew in all ricotta samples, and within the first week, all of them crossed the threshold, which was set to 7 log CFU/g because it was previously observed that yeast proliferation above 6–7 CFU/g can produce important sensory defects [24]. Differences were found between control and treated cheese: control ricotta samples became unacceptable after 5 days, whereas treated cheese remained acceptable after 6–7 days (Table 1). Regarding mesophilic bacteria (data not shown), similar trends to those reported for *Pseudomonas* spp. were recorded.

**Figure 4:**
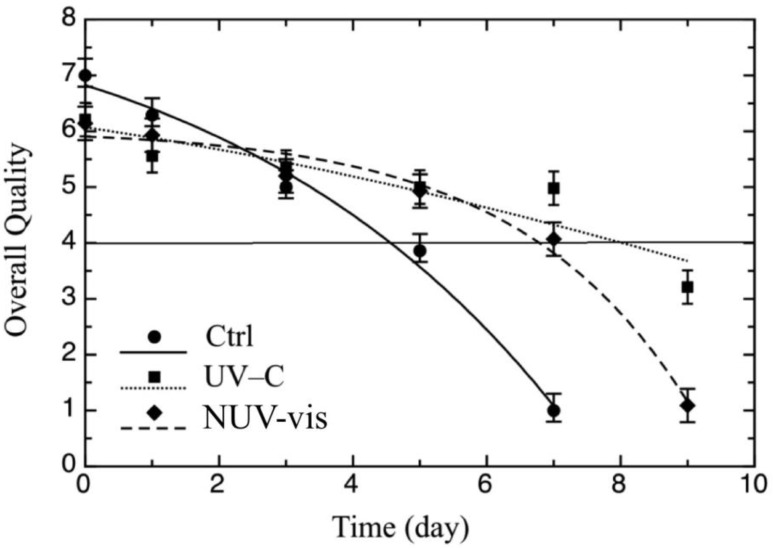
Evolution of sensory quality during the shelf life test of ricotta cheese inoculated with *P. fluorescens* (DSM 50090) (10^3^ CFU/mL), treated with UV-C and NUV–vis light, at one light dose per technology, with respective control samples (Ctrl). A total of 36 samples were treated once, on the same day, which corresponded to time zero of the storage period, and then analyzed for 9 days. The continuous line represents the best fitting of Equation (2) to the experimental data of sensory quality. The horizontal line represents the threshold for overall quality acceptability set to 4.

The effects of the two technologies on the sensory quality of cheese were also evaluated. Results of the panel referring to the overall quality, which represents the mean values of odor, color, and consistency of ricotta cheese, are presented in Figure 4. Two considerations are necessary to underline differences among samples, which are referred to the quality either just after the treatments or during storage. Cheese quality scores immediately after both treatments were slightly lower than for control samples, because panelists detected some off-odors. This off-odor perception in samples treated with UV-C could be attributed to photo-induced changes, e.g., photo reactivity of proteins, which was also attested previously [22], being ricotta a food very rich in amino acids [33]. For NUV–vis-treated samples, these detected off-odors may be due to the fact that sample exposure for 400 s would increase the surface temperature, thus affecting the product characteristics and in particular the smell perceived just after the treatment [34]. However, this initial apparent defect disappeared during storage, and it was possible to observe two different kinetics in terms of acceptability for the treated and untreated samples, over shelf life. Control cheese samples became unacceptable after ~5 days, due to a general product spoilage which mainly affected odor and color [1]. UV-C- and NUV–vis-light treated samples remained acceptable for 7–8 days, with a very similar trend. This would suggest that the application of these light technologies in combination with proper refrigeration and packaging conditions, could contribute to the color, odor, and texture preservation of ricotta cheese for more days. Hyun and Lee [26] also reported that it may be important to find the appropriate conditions of irradiance and storage temperature to minimize sensory quality changes for each individual type of food.

Color is one of the most important quality parameters used to evaluate food quality with light-based technologies [16,35]. However, in the present study, the two types of treatments did not affect cheese color, which was detected sensorially during storage (data not shown). Data from sensory results were also fitted to calculate SAL. The re-parameterized Gompertz equation (equation 2) was used because one of the fitting parameters was SAL, i.e., the number of days necessary to reach the score of 4, which represents the threshold for product acceptability. Results of the fitting procedure of the sensory data are also reported in Table 1, as SAL values of the three samples. Treated cheese recorded general acceptability values higher than those of control samples; in particular, while the SAL value of untreated cheese was 4.5 ± 0.4 days, the values recorded for treated cheese were 2 or 3 days higher.

Both microbiological and sensory fitting values (MAL and SAL) were taken into account for shelf life estimation [36]. Results in terms of shelf life (Table 1) showed that the treated samples had a longer shelf life than the control, in agreement with other previous studies, where the effects of photo-sanitizing technologies on product quality were also evaluated [37,38,39]. In particular, the untreated product became unacceptable after 4 days of storage, whereas treated ricotta recorded a shelf life of more than 6 days. Data also evidenced that UV-C and NUV–vis light are technologies with very comparable efficacy. The preservation effects of these two sanitizing technologies on microbial and sensory quality make them feasible to be used, also considering the intentional inoculation.

## 4. Conclusions

The effects of UV-C and NUV–vis were assessed on contaminated ricotta cheese. The effectiveness of these treatments was compared after similar treatment doses (J/cm^2^). Both microbial and sensory quality were assessed during subsequent refrigerated storage. From the results, it is possible to conclude that both surface-decontaminating technologies affected microbial proliferation and controlled sensory deterioration with comparable efficacy. Therefore, the final shelf lives of inoculated control and treated cheese were significantly different, and in particular, a shelf life extension was recorded for both types of treated cheese. The efficacy recorded in the study would suggest further investigation on industrial ricotta cheese, with the final aim of substituting the traditional thermal treatment with appropriate combinations of novel mild sanitizing technologies. In addition, it is also worth considering that further research could be still carried out, because both techniques could be further optimized to extend ricotta shelf life. Different process parameters, such as distance of the sample from the lamps, sample position in the treatment cell, and treatment time, could be experimentally changed so as to reach an even better shelf life extension. The current results are interesting not only for their novelty but also because the application of these optimized surface techniques is promising for the entire dairy sector and applicable to other fresh products with higher added value than ricotta cheese.

## Figures and Tables

**Table 1:** Microbiological acceptability limit (MAL) and sensory acceptability limit (SAL) measured on a day as fitting parameters of Equations (1) and (2) to microbiological and sensory experimental data, respectively. The goodness of fitting was evaluated by the χ^2^ value. The values of shelf life are the lowest value among the two MAL and the SAL for each sample. Ultraviolet light-treated cheese = UV-C; Near-UV-visible light-treated cheese = NUV–vis; Control sample (inoculated and not treated) = Ctrl.

Samples	MAL^Yeasts^ (day)	MAL^Pseudomonas spp.^ (day)	SAL^Overall Quality^ (day)	Shelf Life (day)
Ctrl	5.82 ± 0.19 ^b^ (χ^2^ = 0.06)	4.46 ± 0.86 ^b^ (χ^2^ = 1.75)	4.50 ± 0.40 ^b^ (χ^2^ = 0.20)	4.46 ± 0.86 ^b^
UV-C	6.53 ± 0.25 ^a^ (χ^2^ = 0.42)	6.23 ± 0.25 ^a^ (χ^2^ = 0.24)	8.02 ± 1.08 ^a^ (χ^2^ = 0.78)	6.23 ± 0.25 ^a^
NUV–vis	6.81 ± 0.17 ^a^ (χ^2^ = 0.15)	6.87 ± 0.45 ^a^ (χ^2^ = 0.45)	6.78 ± 0.42 ^a^ (χ^2^ = 0.30)	6.78 ± 0.42 ^a^

^a,b^ Data in each column with different letters are significantly different (*p* < 0.05).
